# Familial Hemophagocytic Lymphohistiocytosis (FHLH) Perforin Deficiency: A Case Study and Literature Review

**DOI:** 10.7759/cureus.55770

**Published:** 2024-03-08

**Authors:** Badriah G Alasmari, Samah E Mohammed, Mohammedelhassan Ali, Lina Elzubair, Osama A Altayeb, Khalid S Alshehri, Mohammed Alpakra, Mohaned Mohammed, Ali Alabbas

**Affiliations:** 1 Pediatric Hematology and Oncology, Armed Forces Hospital Southern Region, Khamis Mushait, SAU; 2 Pediatrics, Armed Forces Hospital Southern Region, Khamis Mushait, SAU; 3 Pathology, Armed Forces Hospital Southern Region, Khamis Mushait, SAU; 4 Flowcytometry, Flow Cytometry Laboratory for Leukemia & Lymphoma Diagnosis, Khartoum, SDN; 5 Pediatric Intensive Care Unit, Armed Forces Hospital Southern Region, Khamis Mushait, SAU; 6 Pediatrics, Najran General Hospital, Najran, SAU

**Keywords:** prf1, severe sepsis, perforin, familial disease, hemophagocytic lymphohistocytosis (hlh)

## Abstract

Hemophagocytic lymphohistocytosis (HLH) is a severe and fatal immunological disorder that is either primary (i.e., familial) or secondary (i.e., acquired). The primary type comprises autosomal recessive disorders with gene mutations related to natural killer cells and cytotoxic T-cells, whereas the secondary type is related to other pathological causes, such as Epstein-Barr virus, bacterial or fungal infection, autoimmune conditions or autoinflammatory diseases, metabolic disorders, and cancer. In this report, we discuss a 37-day-old male who was brought to the emergency room with fever, decreased activity, and hepatosplenomegaly, with a strong family history of unknown cause of death for three siblings who died at the ages of one to two months. A whole exome sequencing confirmed the clinical diagnosis of familial HLH due to mutation in the PRF1 gene. We note the special importance of genetic counselling and antenatal screening or early neonatal screening in families affected by HLH, as this case highlights the importance of early diagnosis and intervention of primary HLH.

## Introduction

Hemophagocytic lymphohistocytosis (HLH) is a severe, life-threatening, immunological disorder, also known as macrophage activation syndrome, and it is a group of disorders of the immune system that can be triggered by infection, cancer, and rheumatological disease. In HLH, the immune system acts in a dysregulated manner, and the abnormal functioning of white blood cells and cytokines can damage organs, including the liver, spleen, bone marrow, and brain, which can lead to severe organ damage and death [1,2]. The HLH is characterized by clinical and laboratory findings, including fever, hepatomegaly, splenomegaly, pancytopenia, hyperferritinemia, low fibrinogen level, increased triglyceride level, coagulation defect, and multiple organ failure [2]. The immunological cause of HLH is related to cytotoxic cells and natural killer cells, and it is either a primary or secondary type [3]. The primary type is autosomal recessive and divided into five subtypes according to the defective gene: PRF1, STX11, UNC 13D, HPLH, and STXBP2. The PRF1 gene, located on chromosome 10q22.1, codes for perforin, which is responsible for lymphocyte granule-mediated cytotoxicity [4].

HLH has been found to be associated with other conditions, such as combined immune deficiency, chronic granulomatous disease, autoantibody disorders, and antibody deficiency [5]. HLH has proven to be exacerbated by other conditions, such as viral, bacterial, and fungal infections or malignancies, as well as autoimmune disorders [6]. Secondary infection has been shown to cause increased morbidity and mortality [7].

According to the HLH (2004) protocol, treatment options for HLH include chemotherapy, corticosteroids, supportive therapy, and stem-cell transplantation, which is recommended in refractory and relapsing cases [8]. Immunosuppressive therapy is not recommended for patients with severe infections or primary immunodeficiency [9].

## Case presentation

We present a case of a 37-day-old boy who was delivered at term on 11 August 2023 via spontaneous vaginal delivery to first-degree cousins, with an uneventful pregnancy and no neonatal intensive care unit admission. The patient had three siblings, the first one died at the age of one month, and the other two siblings died at the age of two months. The patient’s parents denied any apparent cause of death for their babies, and the patient has three other healthy living siblings. The patient’s previous medical history was unremarkable, and he was in his usual status until 32 days of age when he presented to the emergency room on 16 September 2023 with fever and upper respiratory tract symptoms, decreased activity, and poor suckling. Upon examination, he was hemodynamically stable, with no signs of respiratory distress with equal air entry bilaterally without added sounds, with normal heart sounds and no murmur, and anterior fontanelle flat with normal tone, power, and reflexes, and abdominal examination revealed hepatosplenomegaly without lymphadenopathy.

The patient was admitted to the high-dependency unit of the pediatric ward and started on intravenous antibiotics, with an initial admission diagnosis of fever without focus to rule out sepsis for further workup, and was started on intravenous ceftriaxone empirically. His initial laboratory investigations showed cytopenia, elevated liver enzymes, deranged coagulation profile, elevated inflammatory markers, unremarkable initial capillary blood gas, and elevated lactate dehydrogenase. Polymerase chain reaction for respiratory viral panels detected human rhinovirus/enterovirus. The patient’s anti-Epstein-Barr virus profile was highly positive, with elevated ferritin. A peripheral blood smear showed large, abnormal lymphoid cells, no classical blasts, and moderate thrombocytopenia. Flow cytometry indicated perforin deficiency (Table 1).

**Table 1:** Initial labs showing multiorgan impairment. TWBC = total white blood cell count, ANC = absolute neutrophil count, ALT = alanine transaminase, AST = aspartate aminotransferase, GGT = gamma-glutamyl transferase, ALP = alkaline phosphatase, LDH = lactate dehydrogenase, INR = international normalized ratio, APTT = activated partial thromboplastin clotting time, CRP = C-reactive protein, EBV = Epstein-Barr virus

Lab test	Initial lab result	Lab result after two days	Reference Range
Hemoglobin	12.5 g/dL	2.3 g/dL	10–14 g/dL
TWBC	8.47 x 10^9^/L	4.32 x 10^9^/L	6–17 × 109/L
Platelets	59 x 10^9^/L	17 x 10^9^/L	150–450 × 109/L
ANC	1.19 x 10^9^/L	0.93	1.8–8 × 109 /L
Na	121 mmol/L	173 mmol/L	131–145 mmol/L
K	4 mmol/L	8.1 mmol/L	3.6–5.5 mmol/L
Urea	3.5 mmol/L	6.1 mmol/L	1.79–9.64 mmol/L
Creatinine	40 U/L	73 U/L	27–53 U/L
Total protein	43 g/L	41 g/L	43–69 g/L
Total bilirubin	78.9 μmol/L	55 μmol/L	<34.2 μmol/L
Direct bilirubin	48.4	37	1.7–8.6 µmol/L
Albumin	25 g/L	23 g/L	35–50 g/L
ALT	171 U/L	127 U/L	10–32 U/L
AST	395 U/L	331 U/L	18–63 U/L
GGT	484 U/L	240 U/L	5–40 U/L
ALP	291 U/L	146 U/L	60–321 U/L
LDH	594 mmol/L	-	140–304 mmol/L
Prothrombin	20.1 s	23.9 s	12–15 s
INR	1.69	2.03	<1.4
APTT	75.9 s	93 s	25–42 s
Fibrinogen	136.02 mg/dL	75 mg/dL	150–400 mg/dL
Ferritin	3460 μg/L	-	13.3–191.9 µg/L
Triglycerides	1.28 mmol/L	-	0.45–1.71 mmol/L
CRP	108.8 mg/L	200 mg/L	<10 mg/L
Procalcitonin	5.233 ng/mL	-	<0.1 ng/mL
Flow cytometry	Complete loss of perforin	-	-
Respiratory panel	Human rhinovirus/enterovirus detected	-	Negative
EBV antibodies profile	Highly positive	-	Negative

**Figure 1:**
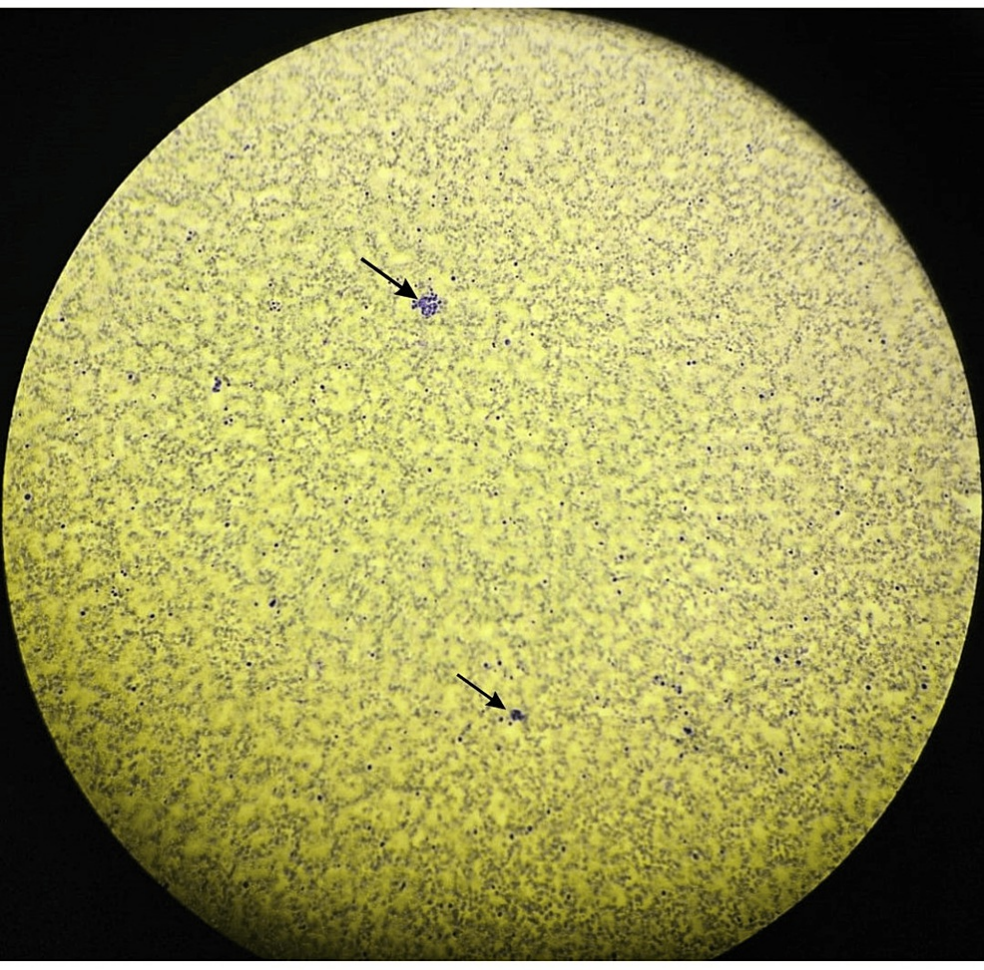
Bone marrow aspirate (Wright-Giemsa stain) x10, showing bloody diluted aspirate with marked hemophagocytosis.

**Figure 2:**
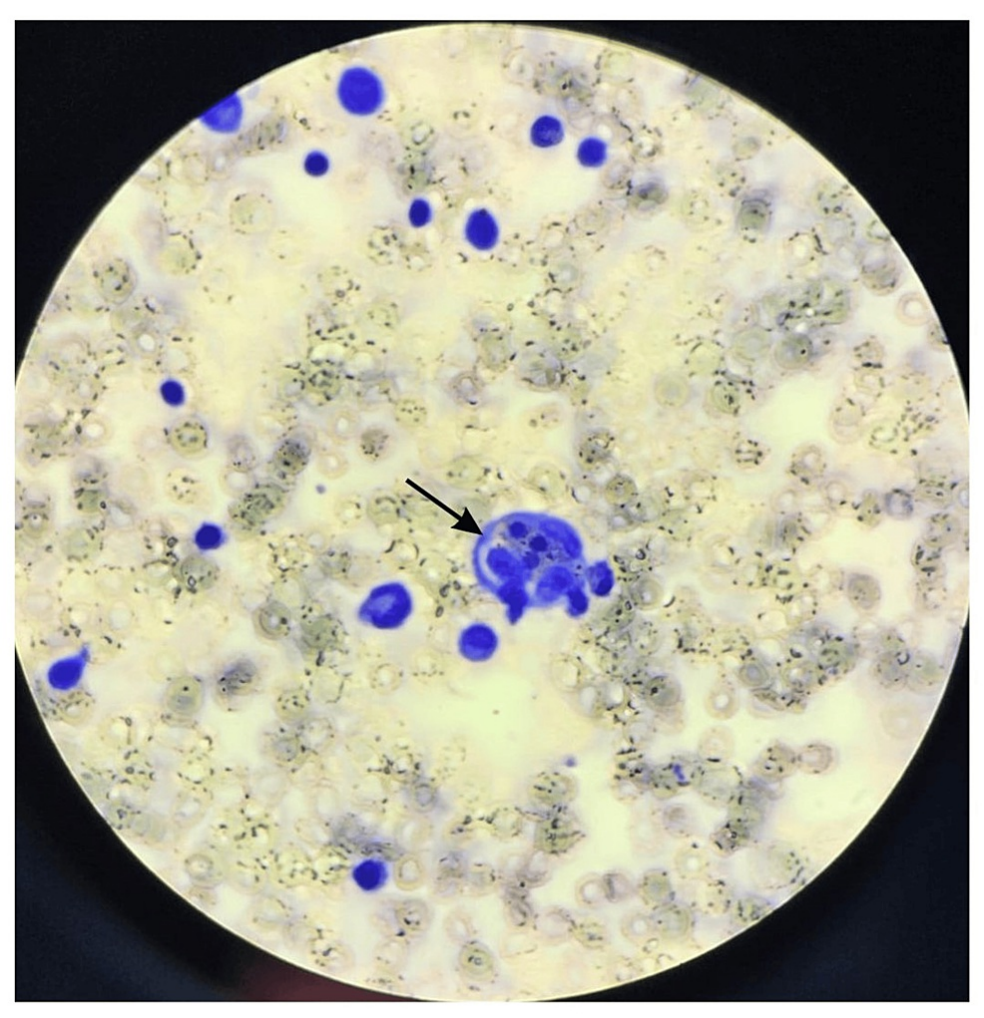
Bone marrow aspirate (Wright-Giemsa stain) x100, showing bloody diluted aspirate with marked hemophagocytosis.

**Figure 3:**
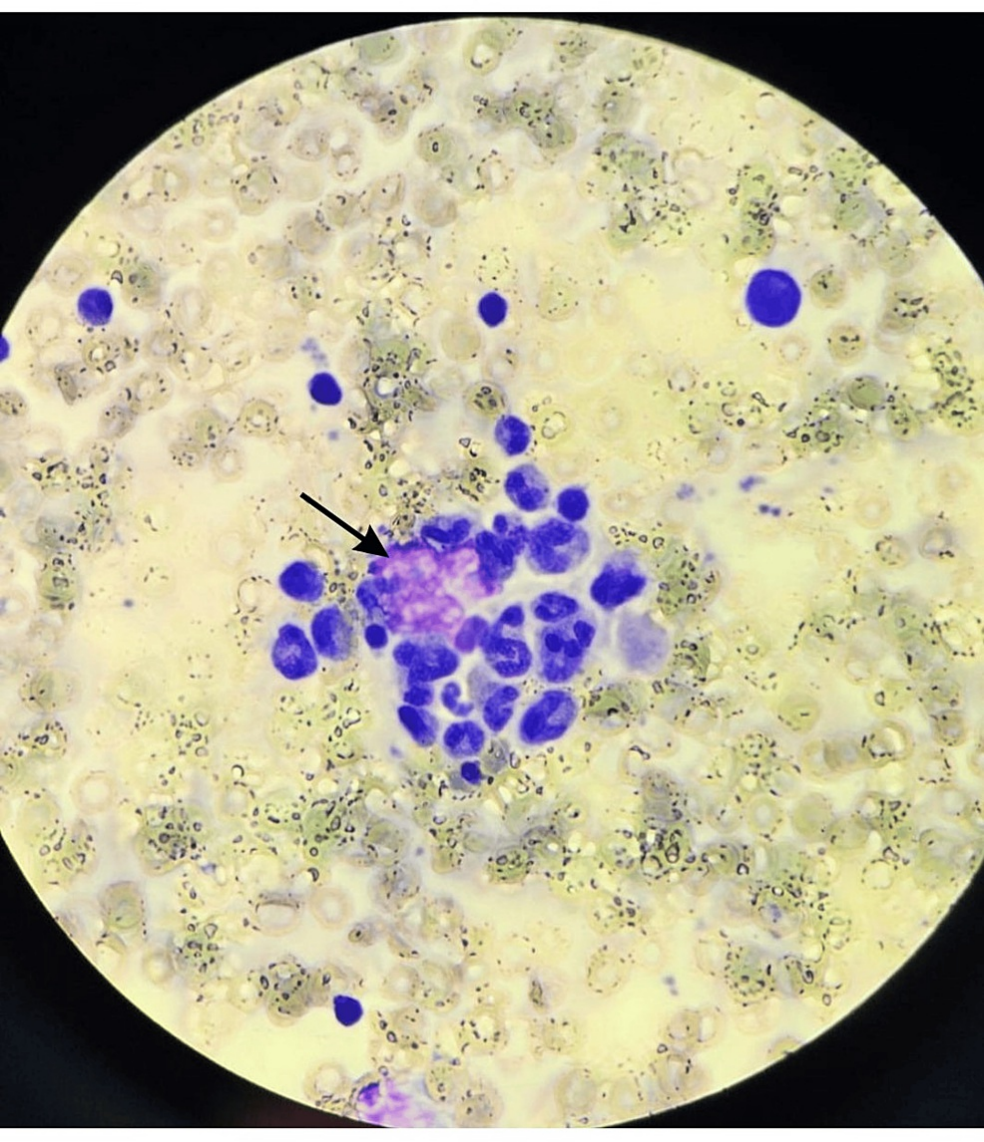
Bone marrow aspirate (Wright-Giemsa stain) x100, showing bloody diluted aspirate with marked hemophagocytosis.

On the second day of admission, the patient was shifted to the pediatric intensive care unit due to decreased level of consciousness. A multidisciplinary team was assigned to the patient, and his antibiotic was upgraded to vancomycin and meropenem. The hematology team proceeded with bone marrow aspiration on the third day of admission as the patient met six criteria for HLH. A bone marrow biopsy was done, revealing cellular bone marrow with marked hemophagocytosis. Immunophenotype results showed no indication of malignant lymphoma or blast cells (Figures 1-3).

The above-mentioned findings were consistent with primary familial HLH type 2. As the patient fulfilled the criteria for HLH, he was treated for primary HLH perforin deficiency (Table 2). A blood sample for whole exome sequence (WES) was performed, and the patient was started on the HLH 2004 treatment protocol with dexamethasone (10 mg/m^2^/day). The patient’s condition deteriorated further as he developed signs and symptoms of shock. Therefore, he was started on inotropic support, intubated, and connected to a mechanical ventilator. The patient became anuric, coagulopathic, and pancytopenic. Supportive management continued while maximizing inotropic support.

**Table 2:** Hemophagocytic lymphohistocytosis laboratory results. Hb = hemoglobin, PLT = platelet count, NK = natural killer cells, WES = whole exome sequence

Lab test	Lab value	Normal value
Hb	2.3 g/dL	10–14 g/dL
PLT	17 x 10^9^/L	150–450 × 109/L
Ferritin	3460 μg/L	13.3–191.9 μg/L
Triglycerides	1.28 mmol/L	0.45–1.71 mmol/L
Fibrinogen	104.3 mg/dL	150–400 mg/dL
Bone marrow aspiration	Cellular bone marrow with marked hemophagocytosis for verification of primary hemophagocytic lymphohistiocytosis.	-
NK activity	Normal	-
Genetic test	PRF1	-
WES	PRF1 NM_001083116.1:c.50de I Frameshift Pathogenic (class 1)	-
Peripheral blood smear	Large, abnormal lymphoid cells, no classical blasts, with moderate thrombocytopenia suggestive of infection and severe thrombocytopenia with normal-sized platelets	-
Flow cytometry	Complete loss of perforin	-

The patient then developed a pulmonary hemorrhage requiring high ventilation. His condition deteriorated further, as he became bradycardic and then developed cardiopulmonary arrest. Resuscitation was done according to the Pediatric Advanced Life Support guidelines, to which the patient did not respond, and death was declared on the fourth day of admission due to septic shock, multi-organ dysfunction, acute respiratory distress syndrome (ARDS), and disseminated intravascular coagulation (DIC). The WES study came back one month later, which identified a homozygous pathogenic variant in the PRF1 gene. The patient’s parents declined a WES study for both them and the patient’s siblings, as they were not planning to have another pregnancy and were satisfied with the size of their family members despite full counselling by the hematology and genetics teams.

## Discussion

HLH is characterized by multisystem inflammation caused by dysregulation of cytotoxic T cells and NK cells [9]. Two different conditions are seen in HLH, genetic and acquired forms [5]. The genetic form of HLH is either an X-linked or an autosomal recessive mode of inheritance and can be divided into familial hemophagocytic lymphohistiocytosis (FHL) and HLH associated with inherited immunodeficiency such as X-linked lymphoproliferative syndrome, Griscelli syndrome, and Chediak-Higashi syndrome. The acquired forms of HLH develop due to profound immunological activation of the immune system due to malignancy or severe infection [6].

HLH is a fatal immunological disorder characterized by fever, pancytopenia, hepatosplenomegaly, and hemophagocytosis in the bone marrow, liver, spleen, and lymph nodes [4]. It is also known as macrophage activation syndrome or hemophagocytic syndrome; in primary-type HLH, the genetic defect causes severe impairment of natural killer (NK) cells and cytotoxic T-cell function, causing ineffective cell-killing associated with increased released cytokines [2].

In a multicenter retrospective study by Xu et al. [10], the median age of children at the time of diagnosis of HLH was 2.2 years, with an incidence of 1:50,000-1:300,000 live births, most of which are born healthy and develop symptoms in the first two to six months of life. Therefore, neonatal screening can play a role in early diagnosis during this window, with better outcomes with early treatment.

In our patient with familial HLH type 2, the immune disorder was related to a mutation of a gene coding for perforin (PRF1), which is essential for cytotoxicity in NK cells and cytotoxic T-cells needed to fight against infectious organisms. Additionally, 20%-50% of familial HLH cases are caused by a mutation in the PFR1 gene [8]. The disease must be differentiated from other serious conditions, such as autoimmune disorders, severe infections, malignancies, immune deficiency syndromes, systemic inflammatory response syndrome, and multiorgan dysfunction [9].

Our patient met all diagnostic criteria for HLH, including fever, splenomegaly, pancytopenia, hypertriglyceridemia, elevated ferritin level, hemophagocytosis, and decreased or absent NK cells, in addition to supportive criteria evidenced by cerebral symptoms, elevated transaminase, and protein and bilirubin levels (Table 3). Our patient started treatment according to the HLH 2004 protocol, but, unfortunately, he did not show any improvement and died on day four of admission [4]. The prognosis of familial HLH in infants is very poor, which is eventually fatal unless hematopoietic stem cell transplantation is performed. The standard treatment protocol includes chemotherapy, corticosteroids, supportive care, and stem transplantation, but despite aggressive treatment, most patients die [9].

**Table 3:** The diagnostic criteria for HLH that were used in the Histiocyte Society HLH-2004 study [4].

(A) Genetic defect consistent with HLH or (B) five out of eight clinical and laboratory criteria fulfilled
Fever
Splenomegaly
Cytopenia in ≥2 cell lineages
Hemoglobin <9 g/dL, in neonates <10 g/dL
Platelet count <100 × 10^3^/mL
Neutrophil count <1 × 10^3^/mL
Hypertriglyceridemia (>265 mg/dL) or hypofibrinogenemia (<150 mg/dL)
Hyperferritinemia (>500 ng/mL)
Soluble CD25 >2400 U/mL (or elevated compared with laboratory-defined normal ranges)
Hemophagocytosis in bone marrow, spleen, lymph nodes, or liver
Low or absent NK-cell cytotoxicity

## Conclusions

Familial HLH is a serious and fatal immunological disorder that is either primary, familial, or secondary caused by other disorders; it requires a high index of suspicion for diagnosis, especially in infants and neonates, as well as differentiation from other serious conditions. Early detection of the disease can help save the life of the patient, which can be done by antenatal screening methods or early neonatal screening for HLH, especially in affected families. This early detection, which suppresses an active immune system with the goal of putting the disease into remission and undergoing an allogeneic stem cell transplant to replace the defective immune system with a healthy one from a donor that can offer patients the best chance for a cure, so we can prepare the patient for bone marrow transplant with the matched related donor as early as possible, accompanied by multidisciplinary team involvement, can decrease the rates of mortality and morbidity. Finally, patients with HLH require family counseling and preimplantation genetic testing.
